# Vincent Cristafalo Award Lecture

**DOI:** 10.1093/geroni/igaa057.3199

**Published:** 2020-12-16

**Authors:** Nir Barzilai, Stephanie Lederman

## Abstract

The Vincent Cristofalo Rising Star Award in Aging Research lecture will feature an address by the 2020 recipient, Sean P. Curran, PhD. This award is given by the American Federation for Aging Research, Inc

